# Body mass index is not associated with lymph node metastasis, infection, and seroma after sentinel lymph node biopsy in cutaneous melanoma

**DOI:** 10.1016/j.jdin.2025.02.003

**Published:** 2025-03-25

**Authors:** Nessr Abu Rached, Fries Leifeld, Eggert Stockfleth, Daniel Myszkowski, Yannik Haven, Falk G. Bechara, Thilo Gambichler

**Affiliations:** a Department of Dermatology, Venereology and Allergology, Skin Cancer Center, Ruhr-University Bochum, Bochum, Germany; b Department of Dermatology, Venereology and Allergology, International Centre for Hidradenitis Suppurativa/Acne Inversa (ICH), Ruhr-University Bochum, Bochum, Germany; c Department of Dermatology and Phlebology, Christian Hospital Unna, Unna, Germany; d Department of Dermatology, Dortmund Hospital gGmbH and Faculty of Health, Witten/Herdecke University, Dortmund, Germany

**Keywords:** epidemiology, lymph node metastasis, melanoma, postoperative infection, postoperative seroma, sentinel lymph node biopsy, skin cancer

*To the Editor:* In cutaneous melanoma (CM), sentinel biopsy is still an important diagnostic tool and common reason for hospitalization. Body mass index (BMI) is an important clinical parameter calculated from height and weight. There is a lack of data on BMI in sentinel biopsy outcome in CM. A study by Almeida Oliveira et al showed that a high BMI was associated with a higher rate of lymph node metastases in CM.^1^ Therefore, we aimed to investigate which factors, in particular BMI, were associated with positive lymph nodes, postoperative seroma, and postoperative infection.

Disease-specific and personal parameters of 1034 melanoma patients were recorded in a retrospective, single-center study (Table I). Parameters were analyzed using descriptive statistics, Mann-Whitney *U*, chi^2^ test, and regression analysis.

**Table I:** Patient’s characteristics, disease-specific, and outcome characteristics of 1034 patients with cutaneous melanoma

Parameters	Values
Sex, *n* (%)	
Female	518 (50.1)
Male	516 (49.9)
Age, median (IQR), y	60 (50-72)
BMI, median (IQR), y	27.1 (23.8-33.1)
Tumor thickness, median (IQR)	1.6 (1.1-2.8)
Presence of ulceration, *n* (%)	228 (22.1)
Localization of the primary CM tumor	
Face and head area, *n* (%)	81 (7.8)
Trunk area, *n* (%)	468 (45.4)
Anogenital area, *n* (%)	21 (2.0)
Extremities, *n* (%)	464 (44.9)
Sentinel biopsy-specific outcome data of 1034 patients with CM	
Outcome of sentinel biopsy, *n* (%)	
Positive (metastases)	232 (22.4)
Negative (no metastases)	784 (75.8)
Lymph nodes cannot be detected or found	18 (1.7)
Capsular invasion of lymph node metastases, *n*/all positive lymph nodes (%)	18 (7.8)
Number of resected lymph nodes, median (IQR)	1 (1-2)
Highest detection rate using gamma probe, median (IQR)	562 (137-679)
Postoperative seroma, *n* (%)	
No	952 (92.1)
Yes	82 (7.9)
Postoperative infection, *n* (%)	
No	952 (92.1)
Yes	82 (7.9)
Primary tumor area	47 (4.6)
Sentinel lymph node area	35 (3.4)
Operation duration in min, median (IQR)	55 (41-74)

*BMI*, Body mass index; *CM*, cutaneous melanoma; *n*, absolute number of patients.

Median tumor thickness was 1.6 mm (IQR: 1.1-2.8 mm), and ulceration was found in 22.1% of patients. Postoperative seromas occurred in 7.9% of patients and postoperative infections were also found in 7.9% of patients (both *n* = 82). Patients with positive lymph nodes had a lower BMI (*P* = .016), more frequent ulceration (*P* = .001), and greater tumor thickness (*P* < .001). Nodular melanoma was also more common in these patients (*P* < .001). Logistic regression analysis showed that greater tumor thickness (odds ratio [OR] = 1.1, 95% CI: 1.03-1.18, *P* = .007) and the presence of nodular melanoma (OR = 1.87, 95% CI: 1.29-2.7, *P* = .001) were significant predictors of positive sentinel lymph nodes (SLNs). In logistic regression analysis, the need for split skin grafting (OR = 2.6, 95% CI: 1.06-6.14, *P* = .037) and the presence of a primary tumor in the lower extremities (OR = 2.9, 95% CI: 1.59-5.23, *P* < .001) were significant predictors of postoperative wound infection. Logistic regression analysis identified the presence of a primary tumor on the lower extremities as a significant predictor of the development of a postoperative seroma (OR = 2.7, 95% CI: 1.21-5.83, *P* = .015). In all regression analyses, BMI was not associated with positive SLN, postoperative infection, or seroma (*P* > 0.5).

Our analysis showed that ulceration, nodular subtype, and tumor thickness were independently associated with a positive lymph node. This is consistent with the findings of Yonick et al, who found that positive sentinel status was associated with ulceration and tumor thickness.^2^^,^^3^ However, novel is the association of nodular subtype with lymph node positivity independent of ulceration and tumor thickness. This may reflect the vertical growth and malignant nature of the nodular type, which increases the likelihood of metastasis.

The rate of postoperative seroma and infection is comparable to that reported in the literature at 7.9%.^4^ It was found that it was not the inguinal SLN biopsy that increased the risk of seroma but the primary tumor in the lower extremity. This may result from the sentinel biopsy and resection in the tumor area destroying the lymphatic vessels, which are all in 1 limb. Limitations of the study include its retrospective, single-center design and the nonextreme range of BMI.

In conclusion, BMI does not influence the outcome of SLN biopsy in CM.

## Conflicts of interest

Dr Abu Rached received funding, travel support, and/or personal honoraria for lectures from Novartis Pharma and Johnson & Johnson that were independent of the work submitted. Dr Bechara has received honoraria for participation in advisory boards, in clinical trials, and/or as a speaker from AbbVie Inc, AbbVie Deutschland GmbH & Co KG, Boehringer Ingelheim Pharma GmbH & Co KG, Novartis Pharma GmbH, UCB Pharma, Incyte Corporation, and JanssenCilag GmbH, and MoonLake. Dr Stockfleth has received lecture fees from Almirall, Leo, Pierre Favre, and Philips. Dr Gambichler has received speakers and/or advisory board honoraria from BMS, Sanofi-Genzyme, MSD, Novartis Pharma, Roche, AbbVie, Almirall, Janssen, Lilly, Pfizer, Pierre Fabre, and Merck-Serono outside the submitted work. Drs Leifeld, Myszkowski, and Haven have no conflicts of interest to declare.
